# *De novo* sequencing and comparative analysis of holy and sweet basil transcriptomes

**DOI:** 10.1186/1471-2164-15-588

**Published:** 2014-07-12

**Authors:** Shubhra Rastogi, Seema Meena, Ankita Bhattacharya, Sumit Ghosh, Rakesh Kumar Shukla, Neelam Singh Sangwan, Raj Kishori Lal, Madan Mohan Gupta, Umesh Chandra Lavania, Vikrant Gupta, Dinesh A Nagegowda, Ajit Kumar Shasany

**Affiliations:** Biotechnology Divison, CSIR-Central Institute of Medicinal and Aromatic Plants, P.O. CIMAP, 226015 Lucknow, U.P India; Metabolic and Structural Biology Divison, CSIR-Central Institute of Medicinal and Aromatic Plants, P.O. CIMAP, 226015 Lucknow, U.P India; Genetics and Plant Breeding Divison, CSIR-Central Institute of Medicinal and Aromatic Plants, P.O. CIMAP, 226015 Lucknow, U.P India; Analytical Chemistry Divison, CSIR-Central Institute of Medicinal and Aromatic Plants, P.O. CIMAP, 226015 Lucknow, U.P India

**Keywords:** Comparative transcriptomics, Chromosome, *Ocimum sanctum*, *Ocimum basilicum*, Phenylpropanoids, Terpenoids

## Abstract

**Background:**

*Ocimum* L. of family Lamiaceae is a well known genus for its ethnobotanical, medicinal and aromatic properties, which are attributed to innumerable phenylpropanoid and terpenoid compounds produced by the plant. To enrich genomic resources for understanding various pathways, *de novo* transcriptome sequencing of two important species, *O. sanctum* and *O. basilicum,* was carried out by Illumina paired-end sequencing.

**Results:**

The sequence assembly resulted in 69117 and 130043 transcripts with an average length of 1646 ± 1210.1 bp and 1363 ± 1139.3 bp for *O. sanctum* and *O. basilicum,* respectively. Out of the total transcripts, 59648 (86.30%) and 105470 (81.10%) from *O. sanctum* and *O. basilicum*, and respectively were annotated by uniprot blastx against *Arabidopsis*, rice and lamiaceae. KEGG analysis identified 501 and 952 transcripts from *O. sanctum* and *O. basilicum*, respectively, related to secondary metabolism with higher percentage of transcripts for biosynthesis of terpenoids in *O. sanctum* and phenylpropanoids in *O. basilicum*. Higher digital gene expression in *O. basilicum* was validated through qPCR and correlated to higher essential oil content and chromosome number (*O. sanctum,* 2n = 16; and *O. basilicum,* 2n = 48). Several CYP450 (26) and TF (40) families were identified having probable roles in primary and secondary metabolism. Also SSR and SNP markers were identified in the transcriptomes of both species with many SSRs linked to phenylpropanoid and terpenoid pathway genes.

**Conclusion:**

This is the first report of a comparative transcriptome analysis of *Ocimum* species and can be utilized to characterize genes related to secondary metabolism, their regulation, and breeding special chemotypes with unique essential oil composition in *Ocimum*.

**Electronic supplementary material:**

The online version of this article (doi:10.1186/1471-2164-15-588) contains supplementary material, which is available to authorized users.

## Background

*Ocimum* L., belonging to family Lamiaceae is one of the best known genus for its medicinal properties and economically important aromatic oils. Some *Ocimum* species are also constituents of Ayurvedic and indigenous medicines. This genus is highly variable and possesses wide range of intra- and inter-specific genetic diversity comprising at least 65 [1] to more than 150 species [2] distributed all over the world. Among these, *Ocimum sanctum* L. (*Ocimum tenuiflorum* L.) and *Ocimum basilicum* L. are the two important species used extensively for their medicinal and industrial importance. *O. sanctum*, known as “the holy basil” is native to Asian tropics [3], whereas *O. basilicum* L. or “the sweet basil” is described to be of African origin as per the Germplasm Resources Information Network [4] of United States Department of Agriculture. While holy basil is revered for its spiritual sanctity and medicinal potential [5], the sweet basil is widely used as culinary herb and for fragrance [6]. Both of the two *Ocimum* species are rich reservoirs of innumerable phytochemicals, which comprises predominantly phenylpropanoids and terpenoids with various medicinal and aromatic properties. Most of these phytochemicals are sequestered in specialized anatomical structures, termed glandular trichomes, on the surface of the aerial parts of the plants [7]. *O. sanctum* is known to possess antibacterial, antianaphylactic, antihistaminic, wound healing, radioprotective, antidiabetic, larvicidal, anti-genotoxic, neuro-protective, cardio-protective and mast cell stabilization activity [8]. The leaves and stem of holy basil contain a variety of biologically active constituents like saponins, flavonoids, triterpenoids, and tannins [9]. Urosolic acid (UA) from *O. sanctum* L. is reported to have cardioprotective effect [10]. Some active phenolics like rosmarinic acid, apigenin, cirsimaritin, isothymusin and isothymonin exhibit antioxidant and anti-inflammatory activities [10]. The most important aroma components are described to be 1, 8 cineole, linalool, methyl chavicol (estragole) and to a lesser degree, eugenol [11]. Similarly, the essential oil of sweet basil (*O. basilicum*) is described to be having antifungal, antimicrobial and insect-repelling activities [12]. *O. basilicum*, contains primarily phenolic derivatives, such as eugenol, methyl eugenol, chavicol, estragole, and methyl cinnamate, often combined with various amounts of linalool [13]. This is also reported to be clinically useful for prevention of stroke, and exhibiting anticarcinogenic, antituberculosis and hypoglycemic activities [14, 15]. Thus, the uses of *Ocimum sp.* for therapeutic purposes in addition to their industrial importance for aromatic properties reinforce the importance of ethno-botanical approach as a potential source of bioactive substances.

Despite spiritual, pharmacological, and industrial importance, very little transcriptomic and genomic data of *Ocimum sp.* is available limiting the studies on biosynthetic pathways of important phytochemicals [7]. National Center for Biotechnology Information (NCBI) shows a record of 312 entries in nucleotide database and 23336 EST sequences of *O. basilicum* compared to only 61 entries in nucleotide database and 108 EST sequences of *O. sanctum.* In recent years, several studies have successfully reported the generation of transcriptome data and its analysis as an effective tool to study gene expression in specific tissues at specific time, and also provide a platform to address comparative genomics for gene discovery in non-model plants for which no reference genome sequences are available [16]. Due to the availability of quick, low cost sequencing [17] and high quality annotation using different assembly tools [18] it has become possible to analyze and understand the genome of non model plant like *Ocimum*. Hence, *O. sanctum* and *O. basilicum* were selected for next generation sequencing (NGS) and analysis with the main objective to establish the basic understanding about genes involved in various pathways and the factors involved in the regulation and channelling of the secondary metabolites like phenylpropanoids and terpenoids. So, leaf transcriptome data of *O. sanctum* (CIM Ayu- eugenol rich variety) and *O. basilicum* (CIM Saumya- methylchavicol rich variety) [19] was generated using paired-end (PE) Illumina NGS sequencing platform and genes involved in phenylpropanoids/ terpenoids biosynthesis pathway were identified. This study also reports EST collection of leaf tissues from *O. sanctum* and *O. basilicum* with a number of differentially expressed cytochrome P450s, transcription factors and pathway genes with probable involvement in differential metabolite biosynthesis in *O. sanctum* and *O. basilicum* leaf tissues. Using these datasets, molecular markers of EST-SSRs were also analyzed to facilitate the marker-assisted breeding of these species. Overall, this data set will be a significant advancement in terms of genomic resources in the diverse *Ocimum* genus.

## Results and Discussion

### Transcriptome sequencing, *de novo*assembly and functional annotation of contigs

**Figure 1:**
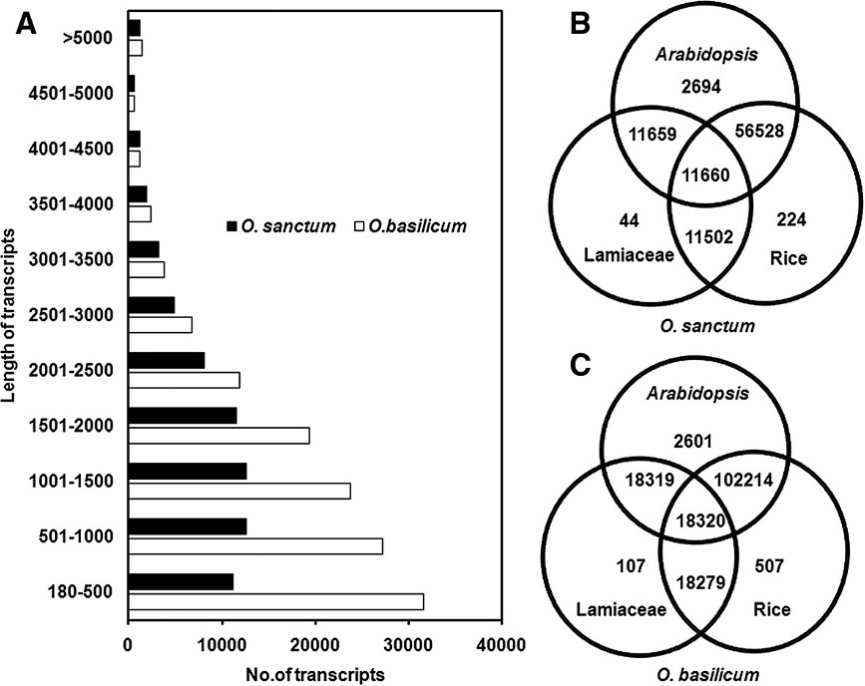
**Transcript abundance and length summary of assembled transcripts. (A)** Length of the assembled transcripts *vs*. Number of transcripts. Venn diagram representing datasets from lamiaceae, *Arabidopsis* and rice databases. **(B)** Number of shared and unique transcripts among lamiaceae, *Arabidopsis* and rice databases in *O. sanctum*. **(C)** Number of shared and unique transcripts among lamiaceae, *Arabidopsis* and rice databases in *O. basilicum.*

In recent years, Illumina sequencing platform has been widely used for transcriptome analysis of plants devoid of reference genomes [20–22]. In order to generate transcriptome sequences, complementary DNA (cDNA) libraries prepared from leaf tissues of *Ocimum* were sequenced using Illumina HiSeq1000 platform. Paired-end Sequencing-by-Synthesis (SBS) yielded raw data of 4.75 Gb and 5.23 Gb for *O. sanctum* and *O. basilicum,* respectively. After filtering and removing adapter sequences from the raw data, 45969831 (45.97 million) and 50836347 (50.84 million) reads comprising of 4542127604 and 5025102762 high quality nucleotide bases for *O. sanctum* and *O. basilicum,* respectively, were retained for further assembly. Filtered reads were assembled into contigs using Velvet assembler at a hash length of 45, which generated 75978 and 290284 contigs for *O. sanctum* and *O. basilicum,* respectively. Transcript generation was carried out using Oases-0.2.08 for the same hash length that resulted in 69117 and 130043 transcripts for *O. sanctum* and *O. basilicum,* respectively. In both cases average contig lengths were of 1646 ± 1210.1 bp and 1363 ± 1139.3 bp with N50 values of 2199 and 1929 in *O. sanctum* and *O. basilicum* respectively (Table 1). The average lengths of transcripts generated using Illumina platform in *Curcuma longa,* cabbage and goosegrass transcriptomes have also been reported with varied lengths of 1304.1 bp, 1419 bp and 1153.74 bp respectively [21–23]. The distribution of assembled transcript length ranged from 180 to >5000 bases. Maximum number of transcripts were of 501–1000 bp size with 12640 transcripts (18.29%) followed by 12613 transcripts (18.25%) of 1001–1500 bp size in *O. sanctum.* Similarly in *O. basilicum*, 180*–*500 bp size transcripts were of highest in number (31594 transcripts, 24.30%) followed by 27208 transcripts (20.92%) of 501–1000 bp size. In both cases, least number of transcripts 591 (0.86%) in *O. sanctum* and 641 (0.49%) in *O. basilicum* were of 4501–5000 bp size (Figure 1A). In root transcriptome of *Ipomoea batatas*, 65.76% unigenes were in the range of 101–500 bp length followed by 20.79% of transcripts of 501–100 bp length [20], similarly in the case of *Medicago sativa*, *Boehmeria nivea*, *Apium graveolens* and *C. longa*, *Centella asiatica* the highest number of transcripts/unigenes were reported with length between 75–500 bp [21–23]. Further, transcripts from both *Ocimum* samples were clustered using CD-HIT-v4.5.4 at 95% identity and query coverage resulting in a total of 130996 transcripts. Blastx search was conducted for assembled sequences of *O. sanctum* and *O. basilicum* against uniprot databases and GO terms were assigned for each unigene based on the GO terms annotated to its corresponding homologue in the uniprot database with the proteins of *Arabidopsis*, rice and lamiaceae family (Table 2; Additional file 1, Additional file 2, Additional file 3). In the case of *O. sanctum*, 59380 transcripts (86%) were annotated with *Arabidopsis*, 56753 (82%) with rice and 11704 (17%) with lamiaceae family whereas 104856 (81%), 102721 (79%) and 18427 (14%) *O. basilicum* transcripts were annotated with *Arabidopsis*, rice and lamiaceae family, respectively. About 442, 694 and 225 transripts of *O. sanctum*; and 107, 2601 and 507 transcripts in *O. basilicum* were uniquely annotated to lamiaceae, *Arabidopsis* and rice databases, respectively (Figure 1B and C). Number of total transcripts annotated from all databases were 59648 (86.30%) and 105470 (81.10%) for *O. sanctum* and *O. basilicum,* respectively.

**Table 1:** **Summary of RNA-Seq**

	***O*** ***.*** ***sanctum***	***O*** ***.*** ***basilicum***
Total Number of HQ Reads	45969831	50836347
Total Number of Reads (Mb) in trimmed data	45.97	50.84
Percentage of HQ Reads in trimmed data	100	100
Total Number of Bases in trimmed data	4542127604	5025102762
Percentage of HQ Bases in trimmed data	97.57	98.47
Percentage of Reads with Non- ATGC Characters in trimmed data	0.67	0.66
Total number of transcripts	69117	130043
Average Transcript Length (bp)	1646.4	1363.5
**N50 value**	2199	1929

**Table 2:** **Annotation summary of** ***O. basilicum*** **and** ***O. sanctum*** **transcripts using Uniprot database**

	UniProt_Lamiaceae		UniProt_***Arabidopsis***		UniProt/rice	
	***O. sanctum***	***O. basilicum***	***O. sanctum***	***O. basilicum***	***O. sanctum***	***O. basilicum***
Total	11704	18427	59380	104856	56753	102721
GO:MF	9449	15109	38618	67205	35303	62227
GO:CC	2402	3126	33480	58087	25602	44215
GO:BP	4460	5966	31720	54533	26758	46351

### Functional classification of *Ocimum*transcriptome by GO

**Figure 2:**
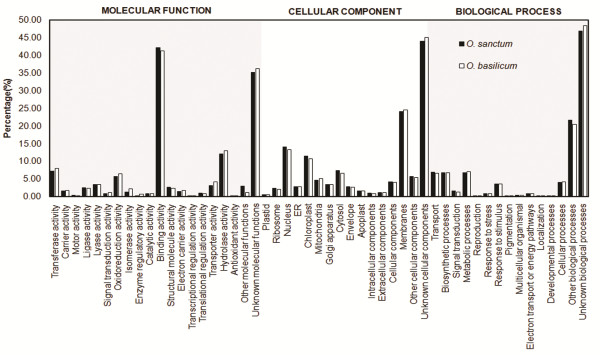
**Histogram of gene ontology classification.** The results are summarized in three main categories: biological process, cellular component and molecular function. Bars represent assignments of *O. basilicum* and *O. sanctum* transcripts (percent) with BLAST matches in the uniprot database (*Arabidopsis*) to each GO term.

Gene Ontology (GO) is an international standardized gene functional classification system offering an updated and a strictly defined concept to comprehensively describe the properties of genes and gene products in any organism [24]. In order to assign putative functions, transcripts from *O. sanctum* and *O. basilicum* were compared against the NR protein sequences of *Arabidopsis*, rice and lamiaceae family available at uniprot database using blastx algorithm. The associated hits were searched for their respective GO. Based on sequence homology, 59380 sequences from *O. sanctum* and 104856 sequences from *O. basilicum* were categorized into 51 functional groups under three main categories: biological process (BP), cellular component (CC) and molecular function (MF) (Figure 2). Highest percentages of genes were classified under ‘unknown groups’ in all the three GO catagories, followed by ‘binding activity’ (42.18% in *O. sanctum* and 43.12% in *O. basilicum*), ‘membranes’ (24.03% in *O. sanctum* and 24.55% in *O. basilicum*), ‘other biological processes’ (21.62% in *O. sanctum* and 20.45% in *O. basilicum*), ‘nucleus’ (13.98% in *O. sanctum* and 13.23% in *O. basilicum*) and ‘hydrolase activity’ (11.99% in *O. sanctum* and 12.94% in *O. basilicum*) were observed. Reports on *Salvia miltiorrhiza* transcriptome, a member of the same family, also represents the ‘binding activity’ of the transcripts in MF category to be with maximum percentage with an anomaly in CC and BP categories [25]. Higher number of genes represented in ‘binding and hydrolase activity’ indicates dominance of gene regulation, signal transduction and enzymatically active processes. Extremely low percentage of genes were classified in terms of ‘antioxidant’ (0.02% both in *O. sanctum* and *O. basilicum*), ‘transcriptional regulation activity’ (0.1% in *O. sanctum* and 0.09% in *O. basilicum*) and ‘localization’ (0.09% in *O. sanctum* and 0.07% in *O. basilicum*) categories (Figure 2). Both the libraries showed similar type of distribution pattern of unigenes under different GO terms. This study suggests the existence of huge potential for new gene identification, as a large number of unigenes from *O. sanctum* and *O. basilicum* were classified to ‘unknown’ subgroups of the three main categories.

### KEGG analysis of *Ocimum*transcriptomes

**Figure 3:**
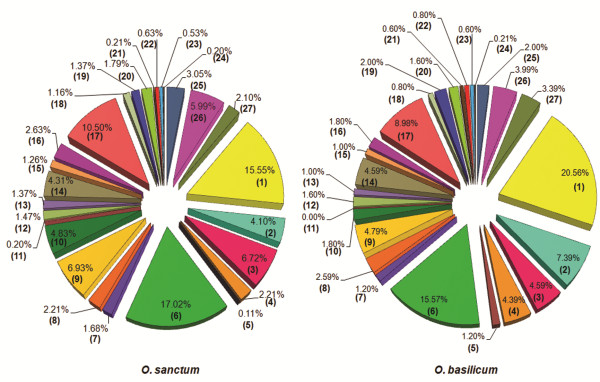
**KEGG classification based on secondary metabolism categories.** Bracketed numbers represent various secondary metabolic pathways abbreviated as: (1) Terpenoid backbone biosynthesis; (2) Streptomycin biosynthesis; (3) Stilbenoid, diarylheptanoid and gingerol biosynthesis; (4) Sesquiterpenoid and triterpenoid biosynthesis; (5) Polyketide sugar unit biosynthesis; (6) Phenylpropanoid biosynthesis; (7) Novobiocin biosynthesis; (8) Monoterpenoid biosynthesis; (9) Limonene and pinene degradation; (10) Isoquinoline alkaloid biosynthesis; (11) Indole alkaloid biosynthesis; (12) Glucosinolate biosynthesis; (13) Geraniol degradation; (14) Flavonoid biosynthesis; (15) Flavone and flavonol biosynthesis; (16) Diterpenoid biosynthesis; (17) Carotenoid biosynthesis; (18) Caffeine metabolism; (19) Butirosin and neomycin biosynthesis; (20) Brassinosteroid biosynthesis; (21) Biosynthesis; of siderophore group nonribosomal peptides; (22) Biosynthesis of ansamycins; (23) Betalain biosynthesis; (24) Anthocyanin biosynthesis; (25) Zeatin biosynthesis; (26) Tropane, piperidine and pyridine alkaloid biosynthesis; (27) Tetracycline biosynthesis.

To identify the biological pathways functional in the leaf tissues of *O. sanctum* and *O. basilicum*, 69117 and 130043 assembled transcripts from both species were mapped to the reference canonical pathways in KEGG. All transcripts were classified mainly under five categories: metabolism, cellular processes, genetic information processing, environmental information processing and others. Highest number of transcripts from both *O. sanctum* and *O. basilicum* were related to metabolism followed by others. In total, all transcripts from *O. sanctum* and *O. basilicum* were assigned to 332 KEGG pathways (Additional file 4). Interestingly, 501 and 952 transcripts, respectively, from *O. sanctum* and *O. basilicum* were found to be involved in biosynthesis of various secondary metabolites. The cluster for ‘Phenylpropanoid biosynthesis [PATH: ko00940]’ and ‘Terpenoid backbone biosynthesis [PATH: ko00900]’ represented the largest group. As observed from Figure 3, the category of ‘terpenoid backbone biosynthesis’ showed highest percentage of transcripts compared to ‘phenylpropanoid biosynthesis’ in *O. sanctum* (20.56%) where as *O. basilicum* had highest percentage (17.02%) of transcripts related to ‘phenylpropanoid biosynthesis’. The list of chemicals and activities specifically in the leaf tissues of *O. sanctum/tenuiflorum* and *O. basilicum* as displayed in the Dr. Duke’s Phytochemical and Ethnobotanical database (http://sun.ars-grin.gov:8080/npgspub/xsql/duke/findsp.xsql?letter=Ocimum&p_request=Go&amt=sc) also supported the higher percentage of terpenoids in *O. sanctum* and phenylpropanoids in *O. basilicum*. From the total compounds in Duke’s database *O. sanctum* showed a higher percentage of diverse terpenoids (53.1%, 34 types) where as *O. basilicum* was found to be rich in phenylpropanoids (65.9%, 27 types; Additional file 5).

### Genes related to biosynthesis of different terpenoids and phenylpropanoids

**Figure 4:**
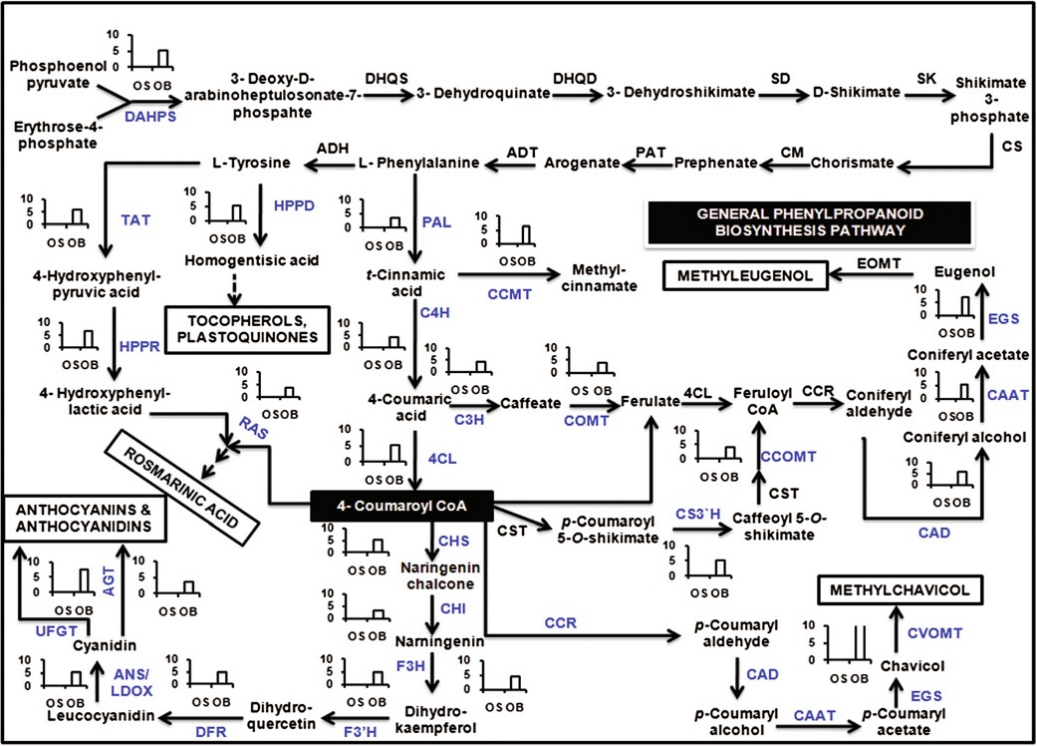
**Phenylpropanoid biosynthetic pathway in** ***Ocimum*** **sps.** Enzymes found in this study are colored in blue. Graphs represent the average log2fold change observed in the digital gene expression analysis. Abbreviations: DAHPS, 3-deoxy-D-arabino-heptulosonate 7-phosphate synthase; DHQS, 3-dehydroquinate synthase; DHQD, 3-dehydroquinate dehydratase; SD, shikimate dehydrogenase; SK, shikimate kinase; CS, chorismate synthase; CM, chorismate mutase; PAT, prephenate aminotransferase; ADT, arogenate dehydratase; ADH, arogenate dehydrogenase; PAL, phenylalanine ammonia lyase; C4H, cinnamate 4-hydroxylase; 4CL, 4-coumarate: CoA ligase; C3H, *p*-coumarate 3-hydroxylase; CS3′H, *p*-Coumaroyl shikimate 3′-hydroxylase; CCMT, cinnamate/*p*-coumarate carboxyl methyltransferase; COMT, caffeoyl O-methyl transferase; CCoAOMT, caffeoyl-CoA O-methyl transferase; CCR, cinnamoyl-CoA reductase; CAD, cinnamyl alcohol dehydrogenase; CAAT, coniferyl alcohol acetyl transferase; EGS, eugenol (and chavicol) synthase; TAT, tyrosine aminotransferase; HPPR, hydroxyphenylpyruvate reductase; HPPD, 4-hydroxyphenylpyruvate dioxygenase; RAS, rosmarinic acid synthase; CHS, chalcone synthase; CHI, chalcone isomerase; F3H, flavanone 3-hydroxylase; F3′H, flavonoid 3′-hydroxylase; DFR, dihydroflavonol 4-reductase; ANS/ LDOX, anthocyanidin synthase; AGT, anthocyanidin 3-O-glucoside 5-O-glucosyltransferase and UFGT, UDP-glucose: flavonoid 7-O-glucosyltransferase.

**Figure 5:**
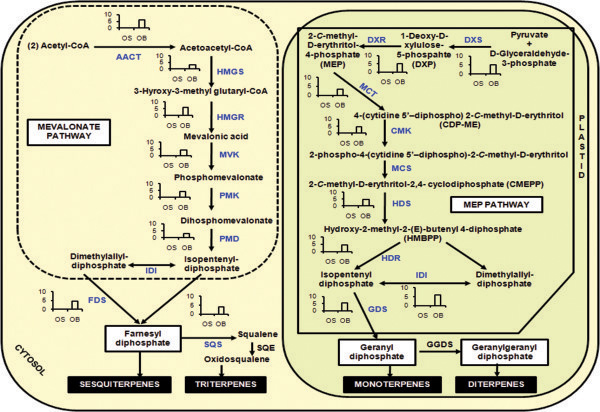
**Mevalonate (MVA) and Non- mevalonate (MEP) biosynthetic pathways in** ***Ocimum*** **sps.** Enzymes found in this study are colored in blue. Graphs represent the average log2fold change observed in the digital gene expression analysis. Abbreviations: AACT, acetoacetyl-CoA thiolase; ADS, amorpha-4,11-diene synthase; ALDH1, aldehyde dehydrogenase 1; BFS, β-farnesene synthase; CPR, cytochrome P450 reductase; CPS, β-caryophyllene synthase; CYP71AV1, amorphadiene-12-hydroxylase; DBR2, artemisinic aldehyde reductase; ECS, *epi*-cedrol synthase; FDS, farnesyl diphosphate synthase; GAS, germacrene A synthase; HMGR, 3-hydroxy-3-methyl-glutaryl coenzyme A reductase; HMGS, 3-hydroxy-3-methyl-glutaryl coenzyme A synthase; IDI, isopentenyl diphosphate isomerase; MVK, mevalonate kinase; PMD, diphosphomevalonate decarboxylase; PMK, phosphomevalonate kinase; SMO, squalene monooxygenase; SQS, squalene synthase; CMK, 4-cytidine 5′-diphospho-2-C-methyl-Derythritol kinase; DXR, 1-deoxy-D-xylulose-5-phosphate reductoisomerase; DXS, 1-deoxy-D-xylulose-5-phosphate synthase; GGDS, geranylgeranyl diphosphate synthase; GDS, geranyl diphosphate synthase; HDR, hydroxy-2-methyl-2-(*E*)-butenyl 4-diphosphate reductase; HDS, hydroxy-2-methyl-2-(*E*)-butenyl 4-diphosphate synthase; IDI, isopentenyl diphosphate isomerase; MCT, 2-*C*-methyl-D-erythritol-4-(cytidyl-5-diphosphate) transferase; MCS, 2-C-methyl-D-erythritol-2,4-cyclodiphosphate synthase (adapted from Olfosson et al. [67]).

**Figure 6:**
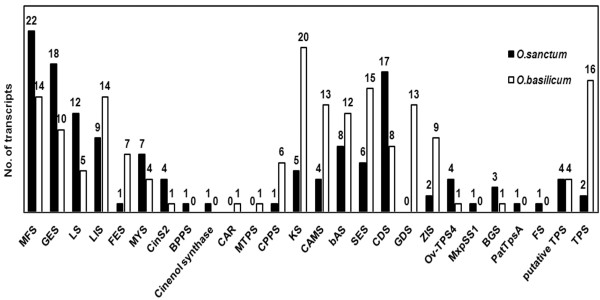
**Transcript abundance of terpene synthases in** ***Ocimum*** **sps.** Abbreviations: Menthofuran synthase (MFS), geraniol synthase (GES), limonene synthase (LS), linalool synthase (LIS), fenchol synthase (FES), myrcene synthase (MYS), 1,8 cineole synthase (CinS2), (+)-bornyl diphosphate synthase (BPPS), cinenol synthase, 3-carene synthase (CAR), monoterpene synthase (MTPS), copalyl diphosphate synthase (CPPS), kaurene synthase (KS), camelliol C synthase (CAMS), beta-amyrin synthase (bAS), selinene synthase (SES), gamma-cadinene synthase (CDS), germacrene-D synthase (GDS), alpha-zingiberene synthase (ZIS), bicyclogermacrene synthase (Ov-TPS4), cis-muuroladiene synthase (MxpSS1), exo-alpha-bergamotene synthase (BGS), gamma-curcumene synthase (PatTpsA), (E)-beta farnesene synthase (FS), putative sesquiterpene synthase (putative TPS) and terpene synthase (TPS).

*O. sanctum* and *O. basilicum* analyzed in this investigation accumulate different types of phenylpropanoids/terpenoids in the essential oil. *O. sanctum* contains mainly eugenol (83.56%), β-elemene (7.47%) and β-caryophyllene (6.93%) [26] whereas *O. basilicum* accumulates methylchavicol (62.54%) and linalool (24.61%) [19]. Precursor molecules for phenylpropanoid biosynthesis are derived from the shikimate pathway (Figure 4) while terpenoid biosynthesis utilizes isoprenoid precursors from cytosolic MVA (mevalonate) as well as plastidial MEP pathways (2-C-methyl-D-erythritol 4-phosphate/1-deoxy-D-xylulose 5-phosphate/non-mevalonate pathways) (Figure 5) [7]. Uniprot annotations against lamiaceae family were used to identify genes encoding enzymes involved in different steps of phenylpropanoid and terpenoid backbone biosynthesis. Both *O. sanctum* and *O. basilicum* annotations comprised of all most all the genes involved in the biosynthesis of essential oil specific phenylproanoids and terpenoids indicating the completeness of transcriptome data (Tables 3, 4 and 5). Higher number of transcripts for *4CL* (4-coumarate: coenzyme A ligase), *ADH* (alcohol dehydrogenase), *TAT* (tyrosine aminotransferase) from phenylpropanoid biosynthetic pathway and *DXS* (1-deoxy-D-xylulose 5-phosphate synthase), GPPS (geranyl diphosphate synthase), and *TPS* (terpene synthase) were detected for terpenoid biosynthetic pathway. The multiplicity of terpenoids produced by a single plant is achieved both by the expression of multiple TPS genes and by the ability of some TPSs to catalyze the production of multiple products [27]. Evidently, annotation of transcriptome data from both *Ocimum* species against *Arabidopsis* and lamiaceae family in uniprot revealed several candidates of probable terpene synthases involved in biosynthesis of terpenoids like- menthofuran, geraniol, limonene, linalool, kaurene, cadinene, selinene, germacrene-D and zingiberene (Figure 6).

**Table 3:** **Transcript abundance in shikimate pathway derived phenylpropanoid biosynthetic pathway genes as per the lamiaceae annotation**

Phenylpropanoid pathway genes	E.C. No.	***Ocimum sanctum***		***Ocimum basilicum***	
		No. of transcripts	Avg RPKM	No. of transcripts	Avg RPKM
Chavicol O-methyltransferase (CVOMT)	2.1.1.146	4	4.55	2	88.55
Eugenol synthase 1 (EGS)	1.1.1.318	6	15.38	8	42.27
*p*-Coumaroyl shikimate 3′-hydroxylase (CS3′H)	1.14.13.36	28	15.72	69	8.96
*p*-Coumarate 3-hydroxylase (C3H)	1.14.13.36	15	8.64	29	4.06
Cinnamate 4-hydroxylase (C4H)	1.14.13.11	7	34.53	21	11.83
4-Coumarate:coenzyme A ligase (4CL)	6.2.1.12	140	9.52	251	5.65
Alcohol acyltransferase (AAT)	2.3.1.84	18	10.05	35	6.12
Cinnamyl alcohol dehydrogenase (CAD)	1.1.1.195	57	15.78	38	24.60
Cinnamoyl-CoA reductase (CCR)	1.2.1.44	78	13.48	112	6.90
Rosmarinic acid synthase (RAS)	2.3.1.140	40	13.59	59	6.28
Phenylalanine ammonia-lyase (PAL)	4.3.1.24	9	91.47	45	11.33
Alcohol dehydrogenase (ADH)	1.1.1.1	101	11.48	226	10.82
Anthocyanidin 3-O-glucoside 5-O-glucosyltransferase (PF3R4)	2.4.1.115	54	11.37	127	3.45
Anthocyanidin synthase (ANS)	1.14.11.19	71	11.47	164	7.45
Cinnamate/p-coumarate carboxyl methyltransferase (CCMT)	2.1.1.-	20	8.12	54	11.01
Caffeoyl CoA O-methyltransferase (CCOMT)	2.1.1.104	16	16.38	36	7.26
Chalcone isomerase (CHI)	5.5.1.6	14	15.92	12	7.51
Chalcone synthase (CHS)	2.3.1.74	29	25.19	72	15.01
Caffeic acid 3-O-methyltransferase (COMT)	2.1.1.68	8	8.97	13	5.03
3-deoxy-D-arabino-heptulosonate 7-phosphate synthase (DAHPS)	2.5.1.54	14	48.28	25	18.98
Dihydroflavonol 4-reductase (DFR)	1.1.1.219	41	12.33	73	7.42
Flavanone 3-hydroxylase (F3H)	1.14.11.9	71	14.76	95	8.55
Flavonoid 3′-hydroxylase (F3′H)	1.14.13.21	47	9.19	72	4.12
Glutathione S-transferase (GST)	2.5.1.18	43	24.55	63	13.80
Hydroxycinnamoyl-CoA shikimate/quinate hydroxycinnamoyltransferase (HSHCT)	2.3.1.133	5	7.05	13	4.53
Hydroxycinnamoyl transferase (HCT)	2.3.1.99	17	12.94	62	3.92
4-Hydroxyphenylpyruvate dioxygenase (HPPD)	1.13.11.27	6	13.73	11	10.70
Hydroxyphenylpyruvate reductase (HPPR)	1.1.1.237	33	7.80	58	7.24
Polyphenol oxidase (PPO)	1.10.3.1	6	50.85	19	44.55
Tyrosine aminotransferase (TAT)	2.6.1.5	63	13.96	101	11.58
UDP-glucose: flavonoid 7-O-glucosyltransferase (UFGT)	2.4.1.91	17	5.33	79	12.14

**Table 4:** **Transcript abundance of MEP pathway derived terpene biosynthetic pathway genes as per the lamiaceae annotation**

MEP pathway genes	E.C. No.	***Ocimum sanctum***		***Ocimum basilicum***	
		No. of transcripts	Avg RPKM	No. of transcripts	Avg RPKM
1-Deoxy-D-xylulose 5-phosphate synthase (DXS)	2.2.1.7	24	15.74	45	15.22
1-Deoxy-d-xylulose 5-phosphate reductoisomerase (DXR)	1.1.1.267	11	15.69	4	50.58
2-C-methyl-D-erythritol 4-phosphate cytidylyltransferase (MCT)	2.7.7.60	3	28.13	7	7.28
4-Diphosphocytidyl-2-C-methyl-D-erythritol kinase (CMK)	2.7.1.148	5	7.73	9	2.66
4-Hydroxy-3-methylbut-2-enyl diphosphate synthase (HDS)	1.17.7.1	2	112.54	4	40.57
Isopentenyl pyrophosphate isomerase (IDI)	5.3.3.2	4	24.58	18	7.18
Geranyl diphosphate synthase (GPPS)	2.5.1.1	15	7.19	21	6.05
Geranylgeranyl diphosphate synthase (GGPPS)	2.5.1.29	8	6.17	7	5.67
Beta-myrcene synthase (MYS)	4.2.3.15	7	6.66	4	7.09
Limonene synthase (LS)	4.2.3.16	12	3.00	5	13.28
Cineole synthase (CinS2)	4.2.3.108	4	8.80	1	12.20
R-linalool synthase (LIS)	4.2.3.26	9	15.11	14	4.37
(−)-Endo-fenchol synthase (FES)	4.2.3.10	1	0.00	7	2.91
Geraniol synthase (GES)	3.1.7.11	18	5.28	10	32.11
Lavandulyl diphsophate synthase (LPPS)	2.5.1.69	14	13.14	10	58.58
Exo-alpha-bergamotene synthase (BGS)	4.2.3.81	3	10.29	1	2.23
Alpha-zingiberene synthase (ZIS)	4.2.3.65	2	3.82	9	12.43
Gamma-cadinene synthase (CDS)	4.2.3.92	8	34.92	17	3.74
Germacrene-D synthase (GDS)	4.2.3.22	0	0.00	13	1.34
Bicyclogermacrene synthase (Ov-TPS4)	4.2.3.100	4	1.87	1	0.91
Selinene synthase (SES)	4.2.3.86	6	1.68	15	7.53
Kaurene synthase (KS)	4.2.3.19	5	1.49	20	1.63
Copalyl diphosphate synthase (CPS)	5.5.1.12	1	0.75	6	1.66
Monoterpene synthase (MTPS)	4.2.3.-	0	0.00	1	0.84
Sesquiterpene synthase (SesqTPS)	4.2.3.-	4	1.43	4	12.81
Terpene synthase (TPS)	4.2.3.-	2	13.13	13	6.83
(+)-Bornyl diphosphate synthase (BPPS)	5.5.1.8	1	0.00	0	10.74

**Table 5:** **Transcript abundance of MVA pathway derived terpene biosynthetic pathway genes as per the lamiaceae annotation**

MVA pathway genes	E.C. No.	***Ocimum sanctum***		***Ocimum basilicum***	
		No. of transcripts	Avg RPKM	No. of transcripts	Avg RPKM
Acetoacetyl-CoA thiolase (AACT)	2.3.1.9	13	14.10	11	26.48
3-Hydroxy-3-methylglutaryl coenzyme A synthase (HMGS)	2.3.3.10	7	11.90	14	2.44
3-Hydroxy-3-methylglutaryl-coenzyme A reductase (HMGR)	1.1.1.34	14	7.04	36	12.17
Mevalonate kinase (MVK)	2.7.1.36	3	4.27	2	6.40
5-Phosphomevalonate kinase (PMK)	2.7.4.2	1	11.56	14	3.46
Mevalonate diphosphate decarboxylase (MDC)	4.1.1.33	9	20.03	12	1.67
Farnesyl diphosphate synthase (FPPS)	2.5.1.10	2	10.42	7	11.62
Squalene synthase (SQS)	2.5.1.21	13	18.30	7	15.75

**Figure 7:**
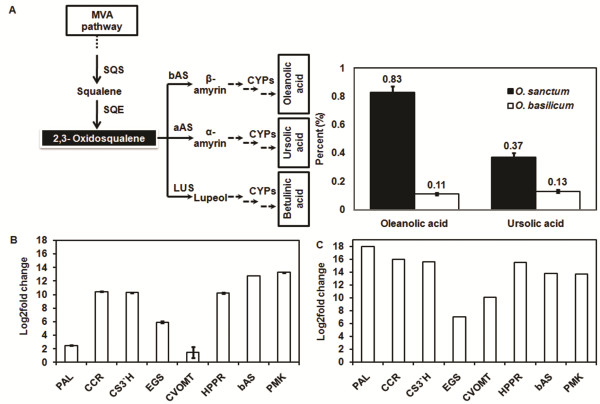
**Data validation using HPLC and Real Time PCR analysis. (A)** Estimation of triterpenoid content in the leaves of *O. sanctum* and *O. basilicum.* **(B)** Validation of the expression pattern of selected pathway genes was carried out using total RNA isolated from *O. sanctum* and *O. basilicum* leaf tissues through quantitative Real time PCR. Error bars represent standard deviation between three replicates. **(C)** Digital gene expression of PAL, CCR, CS3′H, EGS, CVOMT, HPPR, BAS, PMK.

Recently, presence of pentacyclic triterpenoids like ursolic, oleanolic and betulinic acids has been reported in *Ocimum* spp. [28]. This non-aromatic class of compounds have pharmacological importance such as anti-HIV, antibacterial, antiviral, anticancer and anti-inflammatory activities [29]. Like other sesquiterpenoids these triterpenoids also share their origin to farnesyl diphosphate (FDP). FDP is converted to squalene and then to oxidosqualene respectively by squalene synthase (SQS) and squalene epoxidase (SQE) enzymes. Subsequently, oxidosqualene in presence of multifunctional oxidosqualene cyclases (OSCs) *viz.*α-amyrin synthase (aAS), β-amyrin synthase (bAS) or lupeol synthase (LUP) which are then converted to α-amyrin, β-amyrin or lupeol, respectively. OSCs catalyzing the formation of α-amyrin, also produce β-amyrin, finally synthesizing diverse triterpenoids with the help of CypP450s members. Hence, the *bAS* expression cannot be directly correlated with the triterpene accumulation. Similar reports of triterpenoids biosynthesis from these OSCs are available from *Catharanthus roseus* and *O. basilicum* [30, 31]. In this investigation a total of 12 transcripts from *O. basilicum* and 8 transcripts from *O. sanctum* were homologous to β-amyrin synthase as per the *Arabidopsis* annotation. HPLC analysis from the dried leaves of both the *Ocimum* species detected oleanolic and ursolic acids however betulinic acid remained undetected. *O. sanctum* was observed to be having higher content of oleanolic and ursolic acids as compared to *O. basilicum* (Figure 7A).

*Ocimum* spp. is also known to accumulate rosmarinic acid (an ester of caffeic acid and 3,4-dihydroxyphenyllactic acid), which has various pharmacological properties including antioxidant, antibacterial, antiviral and anti-inflammatory activities [32]. Both transcriptomes contained several (32 in *O. sanctum*; 37 in *O. basilicum*) transcripts annotated as rosmarinic acid synthase with average RPKM values of 13.6 and 6.3, respectively. To validate differential digital gene expression, 8 genes were randomly selected for quantitative real time PCR (qPCR). These genes (*PAL*, *CCR*, *CS3′H*, *EGS*, *CVOMT*, *HPPR*, *BAS* and *PMK*) showed up-regulation in *O. basilicum* compared to *O. sanctum* (Figure 7B). All the genes described in this investigation shows up-regulation for *O. basilicum* in digital gene expression results (Figure 7C). This indicates higher accumulation of metabolites in *O. basilicum* compared to *O. sanctum* which is in coherence with the cytological study (Additional file 6). As also discussed earlier, *O. basilicum* is rich in phenylpropanoids with higher content and array of related compounds, which is also in coherence with the observation on upregulation of the phenylpropanoid pathway genes like *PAL*, *CCR*, *CS3′H*, *EGS*, *CVOMT* and *HPPR* in *O. basilicum*.

### Discovery of candidate CYP450s and transcription factors with probable involvement in phenylpropanoid/terpenoid biosynthesis

Cytochrome P450s (CYP450s) are reported to be nature’s most versatile biological catalysts forming the biggest gene families in plants accounting for more than 1% of the total gene annotations in individual plant species [33]. These are generally involved in the biosynthesis of terpenoids, sterols, lignins, hormones, fatty acids, pigments, and phytoalexins in plants [34]. These genes are also the subject of analysis in many of the *de novo* transcriptome sequencing projects in an effort to unravel novel functions of CYPs [24, 25, 35]. Through uniprot annotation against *Arabidopsis*, a total of 386 and 801 transcripts were identified from *O. sanctum* and *O. basilicum,* respectively resembling *CYP*s. However, against lamiaceae family annotation, only 231 transcripts from *O. sanctum* and 542 from *O. basilicum* were identified as members of CYP450 gene family. Out of total *Arabidopsis* database annotated transcripts, 203 transcripts were exclusively annotated to *O. sanctum* and 416 transcripts to *O. basilicum*, whereas 48 and 157 transcripts were found unique to *O. sanctum* and *O. basilicum*, respectively in case of the lamiaceae annotations. Apart from the total and exclusive transcripts, 183 transcripts from *O. sanctum* and 385 transcripts in *O. basilicum* were annotated against both *Arabidopsis* and lamiaceae family in uniprot. All the CYP450s involved in the secondary metabolism were classified under 26 gene families *viz*. CYP51, CYP57, CYP71, CYP72, CYP73, CYP75, CYP76, CYP81, CYP82, CYP84, CYP85, CYP90, CYP91, CYP93, CYP94, CYP95, CYP96, CYP98, CYP706, CYP707, CYP710, CYP711, CYP712, CYP716, CYP721 and CYP734 (Table 6 and 7) with diverse functions in phenylpropanoids and terpenoid metabolism. Among all the CYP families classified, the maximum number of transcripts in both the *Ocimum* sp. belonged to CYP71 family with most abundant CYP71A5 transcripts. Recently, the role(s) of genes of CYP82 and CYP93 families were worked out and described to be involved in flavonoid biosynthesis [36]. Additionally, transcripts of CYP716A class were also identified to be the members of multifunctional oxidases involved in triterpenoids (ursolic, oleanolic and betulinic acids) biosynthesis [37].

**Table 6:** **Numbers of transcripts encoding cytochrome P450s involved in phenylpropanoid metabolism**

	CYP transcripts of ***O. sanctum***		CYP transcripts of ***O. basilicum***		Functions
	***Arabidopsis***annotation	Lamiaceae annotation	***Arabidopsis***annotation	Lamiaceae annotation	
CYP72A14	2	-	8	-	Phenylpropanoid Metabolism
CYP73A1	5	7	23	33	Cinnamate 4-hydroxylase (C4H)
CYP75B1	8	-	8	-	Flavonoid biosynthesis
CYP81D1	2	-	16	-	Phenylpropanoid Metabolism
CYP81F3	1	-	9	-	Phenylpropanoid Metabolism
CYP84A1	2	-	1	-	Coniferaldehyde 5-hydroxylase
CYP93D1	-	-	1	-	Phenylpropanoid Metabolism
CYP98A3	12	-	25	-	4-Coumaryl shikimic/quinic ester 3′-hydroxylase.
CYP98A14	-	16	-	46	*p*-Coumaryl shikimate hydroxylase
CYP707A2	5	-	4	-	Phenylpropanoid Metabolism (abscisic acid 8′-hydroxylase)
CYP707A3	12	-	13	-	Secondary metabolism (abscisic acid 8′-hydroxylase)
CYP710A1	3	-	7	-	Phenylpropanoid Metabolism
CYP711A1	1	-	1	-	Core phenylpropanoid metabolism
CYP712A1	-	-	2	-	Stilbene, coumarine and lignin biosynthesis

**Table 7:** **Numbers of transcripts encoding cytochrome P450s involved in terpenoid metabolism**

	***CYP transcripts of O. sanctum***		CYP transcripts of ***O. basilicum***		Functions
	***Arabidopsis***annotation	Lamiaceae annotation	***Arabidopsis***annotation	Lamiaceae annotation	
CYP51G1	4	-	13	-	Obtusifoliol 14α-demethylase
CYP71A-like	1	-	9	-	(+)-Menthofuran synthase
CYP71B12	-	-	1	-	Biosynthesis of prenyl diphosphates
CYP71B31	1	-	-	-	Mono-/sesqui-/di-terpene biosynthesis
CYP71D13/ D15	-	10	-	16	(−)-Limonene-3-hydroxylase
CYP71D18	-	45	-	43	(−)-Limonene-6-hydroxylase
CYP71 with unknown function	43	99	113	237	Unknown function
CYP72A15	26	-	48	-	Carotenoid biosynthesis
CYP76C3	3	-	8	-	Monoterpene biosynthesis
CYP76C4	-	-	2	-	Mono-/sesqui-/di-terpene biosynthesis
CYP82G1	1	-	2	-	Mono-/sesqui-/di-terpene biosynthesis
CYP85A2	4	-	-	-	Brassinosteroid biosynthesis
CYP90B1	-	-	1	-	Triterpene, sterol, and brassinosteroid metabolism
CYP90C1	2	-	8	-	Steroid biosynthesis
CYP94D2	5	-	6	-	Carotenoid biosynthesis
CYP96A9	1	-	-	-	Mono-/sesqui-/di-terpene biosynthesis
CYP706A7	-	-	4	-	Biosynthesis of steroids
CYP707A4	10	-	14	-	Sterol biosynthesis
CYP716A2	-	-	1	-	Monoterpene biosynthesis
CYP734A1	3	-	1	-	Triterpene, sterol, and brassinosteroid metabolism

**Figure 8:**
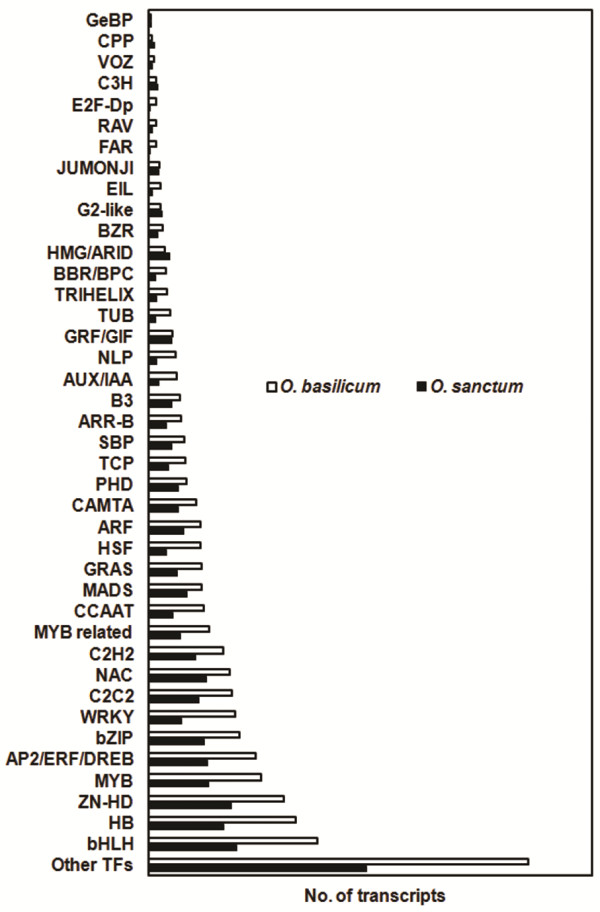
**Distribution of transcripts encoding different transcription factors from** ***O. sanctum*** **and** ***O. basilicum.*** Abbreviations: basic/helix-loop-helix (bHLH), Homeodomain (HB), Zinc finger-Homeobox containing proteins (ZN-HD), MYB, APETELLA 2/Etheylene Responsive factor/Dehydration Responsive Element Binding proteins (AP2/ERF/DREB), basic leucine zipper (bZIP), WRKY, C2C2 [contains DNA binding with one finger (Dof), GATA binding proteins(GATA), Yabby, B-box, Constants-like protein (COL)], (CX2-4CX3FX5LX2HX3-5H)zinc-finger domain containing proteins (C2H2), MYB related, CCAAT binding (CCAAT), MADS- box containing (MADS), SCARECROW (GRAS), Heat Stress Factors (HSF), Auxin Regulatory Factor (ARF), calmodulin binding (CAMTA), PHD type Zinc finger protein (PHD), [TB1(teosinte branched 1), CYC (cycloidea) and PCF family genes] (TCP), Squamosa promoter binding protein (SBP), *Arabidopsis* Response Regulators/ B-motif (GARP-like motif) binding (ARR-B), Auxin induced factors (AUX/IAA), NLP, Growth Regulating factors (GRF/GIF), TUBBY like protein (TUB), trihelix DNA-binding domains (TRIHELIX), Basic Pentacysteine (BBR/BPC), High mobility group (HMG1/2)/ARID/BRIGHT DNA-binding domain-containing protein (HMG/ARID), Brassinosteroid (BR) repressor (BZR), Golden2-like (G2-like), Ethylene-insensitive-like (EIL), Jumonji (jmj)/zinc finger (C5HC2 type) (JUMONJI),FAR, RAV, Cys3His zinc finger domain containing protein (C3H), Vascular Plant Zinc Finger protein (VOZ), Cystein-rich polycomb-like protein (CPP), *GLABROUS1* enhancer-binding protein (GeBP).

Transcription factors (TFs) are sequence specific DNA-binding proteins interacting with the promoter regions of target genes to modulate their expression. In plants, these proteins play a very important role in regulation of plant development, reproduction, intercellular signalling, response to environment, cell cycle and are also important in the modulation of secondary metabolites biosynthesis [38]. In recent years, many studies have been reported on the involvement of various TF families like bHLH, bZIP, Zinc fingers, MYB, ARF, HSF, WRKY, HB and NAC in regulation of secondary metabolites and plant stress responses [25, 39]. As phenylpropanoids and terpenoids are the main determinants of aroma and flavour in *Ocimum*, it becomes important to investigate the transcriptional regulation of the genes involved their biosynthesis, which can further be used to modulate the pathway and develop phenylpropanoid or terpenoid enriched chemotypes. A few transcription factors from other plants, *eg*. EMISSION OF BENZENOIDS I (EOBI), EMISSION OF BENZENOIDS II (EOBII), and ODORANT 1 (ODO 1), MYB4, members of R2R3-MYB family regulate benzenoid/phenylpropanoid volatile biosynthesis in *Petunia hybrida* [40, 41]. ORCA2 and AP2 family member, MYC2, a bHLH family member and WRKY1 regulate indole alkaloid and terpenoid biosynthesis pathway in *Catharanthus roseus* [42, 43]. Similarly, a wound inducible WRKY transcription factor from *Papaver somniferum* was suggested to be involved in benzylisoquinoline biosynthetic pathway [44]. Also, in Lamiaceae family plants like *Salvia miltiorrhiza* and *Perilla frutescens*, TFs belonging to bHLH family are reported to be involved in phenypropanoid biosynthesis pathway [45, 46]. In the present investigation TFs were classified according to uniprot annotation for *Arabidopsis* family. A total of 3489 (5.9%) and 6074 (5.8%) transcripts in *O. sanctum* and *O. basilicum*, respectively were grouped into 40 TF families (Figure 8). Those which were annotated to have sequence specific transcription factor activity but cannot be grouped among any family were included in ‘other’ TFs category, following *Arabidopsis* transcription factor database (http://Arabidopsis.med.ohio-state.edu/AtTFDB/) and Plant transcription factor database (http://planttfdb.cbi.pku.edu.cn/) [47] classification. A systematic analysis of these transcription factors would help in understanding differential regulation of terpenoid and phenypropanoid pathways.

### Cytogenetic characterization of *O. sanctum*and *O. basilicum*

There have been discrepancies regarding the chromosome number of *Ocimum* in literature. Darlington and Wylie [48] and Mehra and Gill [49] considered x = 8 as basic chromosome number for the genus *Ocimum* as a whole, while some other authors suggested that *Ocimum* species are characterized by the different basic chromosome numbers x = 8, 10, 12, or 16 [50]. In order to establish the actual chromosome numbers for the two varieties used in this study, fast growing roots emerging from stem cuttings were examined for somatic chromosome number. Observations recorded from root-tip mitosis reveal somatic chromosome count of 2n = 16 for *O. sanctum* and 2n = 48 for *O. basilicum* and chromosome size below 1 μm (Additional file 6). As the essential oil of the genus *Ocimum* is the reservoir of secondary metabolites, there may be a probable correlation between the chromosome numbers of species and its essential oil yield, which may in turn be affected by expression of related genes. Indeed, DGE and real-time expression analyses showed higher expression of pathway genes in *O. basilicum* compared to *O. sanctum* (Figures 4, 5, 7). Moreover, the ploidy level has been shown to enhance the accumulation of secondary metabolites in *Cymbopogon* [51]. As reported earlier, *O. basilicum* (var: CIM-Saumya) shows more vigorous growth and higher oil content (0.99%) compared to *O. sanctum* (var: CIM-Ayu) with 0.70% oil content [19, 26].

### Analysis of GC content and identification of SSR Markers

Next generation sequencing also offers an opportunity for the analysis of GC content among transcripts and expands the scope for molecular markers such as SSRs. GC content is an important indicator of the genomic composition including evolution, gene structure (intron size and number), gene regulation and stability of DNA [52]. Average GC contents of *O. sanctum* and *O. basilicum* transcripts were analyzed to be 47.12% and 46.39%, respectively (Additional file 7), which is in the range of GC levels of coding sequences in dicots (44-47%) [53]. Simple sequence repeats (SSRs) markers have proven to be valuable tools for various applications in genetics and breeding for the better understanding of genetic variation. As described, more than 150 species [1, 2] of *Ocimum* are reported around the world and hence, polymorphic SSR markers are important for investigations related to genetic diversity, relatedness, evolution, linkage mapping, comparative genomics and gene-based association studies. Transcriptome SSR markers also exhibit high inter-specific transferability [54]. Genus *Ocimum* is highly prone to cross pollination and hence the seed raised population will have variability in metabolite content [10]. The identification of SSRs in *Ocimum sp.* will help in distinguishing closely related individuals and will also provide useful criteria for enriching and analyzing variation in the gene pool of both the plants. Even though SNPs serve as excellent markers especially for high-throughput mapping and studying complex genetic traits, SSRs provide a number of advantages over other marker systems. SSRs with their moderate density still serve as the best co-dominant marker system for construction of framework linkage maps [55]. The transcripts from the data of present investigation were also found to have abundant SSRs. Out of 69117 and 130043 transcripts of *O. sanctum* and *O. basilicum*, 27.77% transcripts (19191) from *O. sanctum* and 17.79% (23141) transcripts from *O. basilicum* were observed to be having SSRs (Table 8 and Additional file 8). The total number of SSR containing sequences in *O. sanctum* and *O. basilicum* were 26232 (37.95%) and 28947 (22.26%), respectively. Following the criteria used to identify these SSRs, di-nucleotide repeats were highest in number for both the species (14.64% in *O. sanctum* and 6.94% in *O. basilicum*), while penta-nucleotide repeats were of lowest occurrence (0.16%) in *O. sanctum* and hexa-nucleotide repeats (0.08%) in *O. basilicum*. The most prevalent dinucleotide SSRs group had the highest occurrence of CT, TC, AG and GA repeats followed by trinucleotide (7.03%) SSRs in *O. sanctum*, while in *O. basilicum* TC, CT, AG and GA dinucleotide repeats were highest. Interestingly, several SSR motifs were linked with unique sequences encoding enzymes (*e.g*. COMT, HPPR, HPPD, PPO, HSHCT, CinS2, ZIS, BGS, LPPS, CDS, MYS, LIS, AAT2, IDI, HDS, DXR, SQS, AACT) involved in terpenoid/phenylpropanoid biosynthesis (Additional file 9). Maximum number of SSRs was observed in *4CL* transcripts of *O. sanctum* where as SSR number was abundant in *ANS* transcripts of *O. basilicum*. The gene specific identification of SSRs in both the *Ocimum* sp. will help in distinguishing closely related individuals and will also provide useful criteria for enriching and analyzing variation in the gene pool of the plant. Similarly, mining of SNPs from NGS-generated transcripts mainly involves clustering and assembling the sequence reads, followed by SNP identification by means of *in silico* approaches [56]. In this investigation, a total of 3245 (66.16%) transitions and 1660 (33.84%) transversions were observed by the SNP finder tool with *O. sanctum* as anchor (Table 9 and Additional file 10).

**Table 8:** **Statistics of SSRs identified from** ***O. basilicum*** **and** ***O. sanctum*** **leaf transcriptome data**

	***O. sanctum***	***O. basilicum***
Total number of sequences examined	69117	130043
Total size of examined sequences (bp)	113791599	177312343
Total number of identified SSRs	26232	28947
Number of SSR containing sequences	19191	23141
Number of sequences containing more than 1 SSR	5128	4383
Number of SSRs present in compound formation	2301	2091
Di-nucleotide repeat	10118	9025
Tri-nucleotide repeat	4859	6029
Tetra-nucleotide repeat	314	363
Penta-nucleotide repeat	109	115
Hexa-nucleotide repeat	223	107

**Table 9:** **Single nucleotide polymorphism (SNPs) statistics**

Summary of SNPs statistics	Number	Percentage (%)
**Tot. no. of Transitions**	**3245**	**66.16**
A < − > G Transitions	1602	32.66
C < − > T Transitions	1643	33.50
**Tot. no. of Transversions**	**1660**	**33.84**
A < − > T Transversions	538	10.97
G < − > T Transversions	363	7.40
C < − > G Transversions	369	7.52
A < − > C Transversions	390	7.95
**Tot. no. of SNPs**	**6565**	

## Conclusion

Terpenoids and phenylpropanoids are the predominant secondary metabolites in *Ocimum* species. These metabolites are synthesized through metabolic divergence from the mevalonate/non-mevalonate and shikimate pathways, respectively, and accumulate in the specialized glandular trichomes on the leaves [7]. So, this study was undertaken with the objective of enriching the existing limited set of genomic resources in *Ocimum*, and to provide a comparative analysis of transcriptomes of two *Ocimum* species having contrasting essential oil composition. To this end, high quality transcriptome database was established for *O. sanctum* and *O. basilicum* by using NGS technology. This is the first report of a comprehensive transcriptome analysis of *Ocimum* species. Genes encoding pathway enzymes related to aromatic components such as volatile terpenoids, phenylpropanoids and non-volatile medicinal compounds such as pentacyclic triterpenes and rosmarinic acid were identified in the transcriptome database; indicating the importance of exploring *Ocimum* species as a source of both medicinal and aromatic compounds. Moreover, several putative CYPs and transcription factors with probable involvement in the biosynthesis and regulation of terpenoids and phenylpropanoids were identified. Further investigations on these putative CYPs and TFs may reveal the reasons behind differential accumulation of phenylpropanoids/terpenoids, along with the similarity/difference in biosynthetic pathways operating in different species of *Ocimum*. Additionally, several SNPs and SSRs were identified in both the transcriptomes which will assist in breeding of *Ocimum* for developing distinct chemotypes. Overall, *Ocimum* transcriptome databases presented here, both individually and collectively, can be exploited to characterize genes related to phenylproanoid and terpenoid metabolism and their regulation, as well as for breeding chemotypes with unique essential oil composition in this largely cross-pollinating species.

## Methods

### Plant material, library preparation and sequencing

Leaf tissues of *O. sanctum* L. (var: CIM Ayu) and *O. basilicum* L. (var: CIM Saumya) were collected from three month old plants grown in the experimental farm at the Bangalore Resource Centre of CSIR-Central Institute of Medicinal and Aromatic Plants. TRIzol method was used for RNA isolation from the leaf tissues. The quality and quantity of total RNA was calculated with a Bioanalyzer (Agilent Technologies, Palo Alto, CA, USA); high-quality (RNA Integrity Number >7) RNA was used. The cDNAs were amplified according to the Illumina RNA-Seq protocol and sequenced using the Illumina HiSeq1000 system, producing 45.97 and 50.84 Mbp of 100-bp paired-end reads for *O. sanctum* and *O. basilicum* respectively. Transcriptome library for sequencing was constructed according to the Illumina TruSeq RNA library protocol outlined in “TruSeq RNA Sample Preparation Guide” (Part # 15008136; Rev. A; Nov 2010). Enriched Poly-A RNA (1 μg) using RNA Purification Beads was fragmented for 4 minutes at elevated temperature (94°C) in the presence of divalent cations and reverse transcribed with Superscript III reverse transcriptase by priming with Random Hexamers (Invitrogen, USA). Second strand cDNA was synthesized in the presence of DNA polymerase I and RNaseH. The cDNA was cleaned up using Agencourt Ampure XP SPRI beads (Beckman Coulter, USA) followed by ligation of “Illumina Adapters” to the cDNA molecules, after end repair and addition of “A”- base. Following SPRI cleanup after ligation, the library was amplified using 11 cycles of PCR, for enrichment of adapter ligated fragments. The prepared library was quantified using Nanodrop and validated for quality by running an aliquot on High Sensitivity Bioanalyzer Chip (Agilent).

### *De novo*assembly and sequence clustering

Raw reads obtained after sequencing were subjected to adapter, B-block and low quality base filtering to obtain the processed reads. *De novo* assembly of the processed reads was carried out using Velvet_1.2.10 for different hash lengths (45–73) [57]. Velvet takes in short reads and assembles them into contigs using paired-end information. This assembly was used by “observed-insert-length.pl” and “estimate-exp_cov.pl” (from Velvet package) to estimate insert length and expected coverage parameters, which were then used to generate a final assembly. The resulting contigs were assembled into transcripts by Oases-0.2.01 for the same (45–73) hash lengths [58], using the assembly from Velvet and clustering them into small groups (loci). It then uses paired end information to construct transcript isoforms. Transcript assembly was selected for the best hash length based on the assembly statistics and the transcripts from both the samples were clustered together using CD-HIT-v4.5.4 at 95% identity and 95% query coverage [59]. The transcriptome data for both the species was submitted to the NCBI under SRA Study accession number SRP039008 for *O. sanctum* and SRP039533 for *O. basilicum*).

### Sequence annotation and functional characterization

Assembled transcripts were blasted against UniProt databases and GO (Gene Ontology) terms were assigned for each unigene based on the GO terms annotated to its corresponding homologue in the UniProt database with the proteins of *Arabidopsis*, Rice and Lamiaceae family. Each annotated sequence may have more than one GO term, assigned either for different GO categories (Biological Process, Molecular Function and Cellular Component) or in the same category [60]. To gain an overview of gene pathway networks, the assigned polypeptides encoded by unigenes from *O. sanctum* and *O. basilicum* transcriptome were mapped to metabolic pathways according to the Kyoto Encyclopedia of Genes and Genomes (KEGG) [61]. The output of KEGG analysis includes KEGG orthology (KO) assignments using KEGG automated annotation server, KAAS (http://www.genome.jp/kaas-bin/kaas_main?mode=partial).

### Read mapping and transcript abundance measurement

RPKM (Reads Per Kilobase per Million) measurement is a sensitive approach by which expression level of even poorly expressed transcripts can be detected using read count as the fundamental basis. For RPKM measurement, reads were first aligned using “Bowtie tool” [62] and “Awk scripting” was used to generate the read count profile from the output file (.sam) of Bowtie alignment. RPKM values were calculated applying the approach adopted by Mortazvi and co-workers [63], to measure the expression level of each assembled transcript sequence. The clustered transcripts were used as the master reference for carrying out the digital gene expression (DGE) analysis by employing a negative binomial distribution model (DESeq v1.8.1 package (http://www-huber.embl.de/users/anders/DESeq/) [64].

### Cytological analysis

Stem cuttings of the *O. sanctum* (var. CIM Ayu) and *O. basilicum* (var. CIM Saumya) were transplanted in moist sand. The fast growing 1 cm long young roots emerging from the stem cuttings were excised and pre-treated for 2.5 h in saturated aqueous solution of *p*-dichloro benzene at 12–14°C, washed thoroughly in water and quickly transferred to Carnoy's mixture (6:3:1) for fixation overnight at room temperature. Next day the fixed roots were transferred to 45% acetic acid for 10 minutes, and thereafter stained in 2% acetocarmine for 2 hrs at 60°C and then overnight at room temperature. The stained root tips were squashed in 45% acetic acid and permanent chromosome preparations were made by removing the cover glass by quick-freeze method followed by dehydration in tertiary butyl alcohol series and mounting in DPEX.

### Real-time PCR analysis

Total RNA was isolated from both *O. sanctum* and *O. basilicum* leaves of same stage and cDNAs were prepared using RevertAid first strand cDNA synthesis Kit (ThermoScientific, USA). Expression of selected pathway genes and cytochrome P450s was analyzed through qPCR using Fast Real Time PCR System (7900HT Applied Biosystems, USA) and Maxima SYBR Green PCR Master Mix (2X) (ThermoScientific, Waltham MA, US) to validate Illumina sequencing data. Each PCR reaction was set up in 15 μl volume containing 7.5 μl of Maxima SYBR Green PCR master mix, 50 ng of cDNA sample prepared using RevertAid first strand cDNA synthesis Kit (ThermoScientific) and gene-specific primers (Additional file 11). The specificity of the reactions was verified by melting curve analysis with the thermal cycling parameters: initial hold (50°C for 2 min); initial denaturation (95°C for 10 min); and 40 amplification cycles (95°C for 15 s; and 60°C for 1 min) followed by additional steps (60°C for 15 s, 95°C for 15 s and 37°C for 2 min). Relative mRNA levels were quantified with respect to the reference gene ‘*actin*’ of *O. sanctum* (SO_2009_transcript16212) [65]. Sequence Detection System (SDS) software version 2.2.1 was used for relative quantification of gene transcripts using the ΔΔCQ method. Threshold cycle (Cq) values obtained after real-time PCR were used for calculation of the ΔCq value (target-reference). The quantification was carried out by calculating ΔΔCq to determine the fold difference in gene expression [ΔCq target – ΔCq calibrator]. The RQ was determined as 2 ^–ΔΔCQ^. All the experiments were repeated using three biological replicates and the data were analyzed statistically (±Standard Deviation).

### Estimation of triterpenoid content

Methanolic extract of 0.5 g dried leaf powder was used for estimation of triterpenoids mainly oleanoleic, ursolic and betulinic acids. HPLC was performed as per previously reported method with slight modification [66] with an instrument (Shimadzu, Japan), consisting of an analytical column (Waters Spherisorb ODS-2, 250 × 4.6 mm, 10 μm), pumps (LC-10AT), autoinjector (SIL-10 AD) and PDA (SPD-M10A). Mobile phase composition used was acetonitrile– water containing 0.1% trifluoroacetic acid (TFA) (85:15 v/v) at a flow rate of 1.0 mL min^−1^. The quantitation was performed at 204 nm as reported earlier.

### Identification of simple sequence repeats (SSRs) and single nucleotide polymorphism (SNPs)

All the transcripts of *O. sanctum* and *O. basilicum* were analyzed with a microsatellite program, MISA (http://pgrc.ipkgatersleben.de/misa/) for identification of SSR motifs having mononucleotide to hexanucleotide repeats. The parameters used for simple sequence repeats (SSRs) were, at least 6 repeats for di- and 5 for tri-, tetra, penta- and hexa- nucleotide. Transitions and transversions identification between *O. sanctum* and *O. basilicum* was carried out using SNPs Finder tool taking *O. sanctum* as anchor (http://snpsfinder.lanl.gov/).

## Electronic supplementary material

Additional file 1: **Transcript annotation of** ***O. sanctum*** **and** ***O. basilicum*** **with Uniprot against** ***Arabidopsis*** **.** (XLSX 14 KB)

Additional file 2: **Transcript annotation of** ***O. sanctum*** **and** ***O. basilicum*** **with Uniprot against Rice.** (XLSX 155 KB)

Additional file 3: **Transcript annotation of** ***O. sanctum*** **and** ***O. basilicum*** **with Uniprot against lamiaceae family.** (XLSX 12 KB)

Additional file 4: **Pathway assignment of** ***O. sanctum*** **and** ***O. basilicum.*** **unigenes based on Kyoto Encyclopedia of Genes and Genomes (KEGG).** (PDF 124 KB)

Additional file 5: **List of chemicals in the leaf tissues of** ***O. sanctum/ tenuiflorum*** **and** ***O. basilicum*** **as displayed in the Dr. Duke’s Phytochemical and Ethnobotanical database.** (XLSX 497 KB)

Additional file 6: **Somatic chromosome preparations of (a)** ***O. basilicum*** **(2n = 48) and (b)** ***O. sanctum*** **(2n = 16) on a scale of 1 μm.** (PDF 111 KB)

Additional file 7: **Base composition report of** ***O. sanctum*** **and** ***O. basilicum.*** (PDF 138 KB)

Additional file 8: **Details of SSRs analysis from the transcripts of** ***O. sanctum*** **and** ***O. basilicum.*** (XLSX 3 MB)

Additional file 9: **List of phenylpropanoid and terpenoid pathway genes containing SSRs identified from the transcripts of** ***O. sanctum*** **and** ***O. basilicum.*** (XLSX 3 MB)

Additional file 10: **Details of SNPs analysis from the transcripts of** ***O. sanctum*** **and** ***O. basilicum.*** (XLSX 13 KB)

Additional file 11: **List of primers used in Real-Time PCR.** (XLSX 3 MB)

## References

[1] Paton A, Harley RM, Harley MM, Holm Y, Hiltunen R (1999). *Ocimum*- an overview of relationships and classification. Medicinal and Aromatic Plants-Industrial Profiles.

[2] Sahoo Y, Pattnaik SK, Chand PK (1997). In vitro clonal propagation of an aromatic medicinal herb *Ocimum basilicum* L. (sweet basil) by axillary shoot proliferation. In Vitro Plant.

[3] **GRINa** http://www.ars-grin.gov/cgi-bin/npgs/html/taxon.pl?25491#dist

[4] **GRINb** http://www.ars-grin.gov/cgi-bin/npgs/html/taxon.pl?25478

[5] Singh N, Hoette Y, Miller R (2002). Tulsi- The Mother Medicine of Nature.

[6] Makri O, Kintzios S (2008). *Ocimum* sp. (Basil): Botany, Cultivation, Pharmaceutical Properties, and Biotechnology. J Herbs Spices Medicinal Plants.

[7] Iijima Y, Davidovich-Rikanati R, Fridman E, Gang DR, Bar E, Lewinsohn E, Pichersky E (2004). The biochemical and molecular basis for the divergent patterns in the biosynthesis of terpenoids and phenylpropanoids in the peltate glands of three cultivars of basil. Plant Physiol.

[8] Rahman S, Islam R, Kamruzzaman AK, Jamal AHM (2011). *Ocimum sanctum* L.: A review of phytochemical and pharmacological profile. Am J Drug Discov Dev.

[9] Jaggi RK, Madaan R, Singh B (2003). Anticonvulsant potential of holy basil, *Ocimum sanctum* Linn, and its cultures. Indian J Exp Biol.

[10] Pattanayak P, Behera P, Das D, Panda SK (2010). *Ocimum sanctum* Linn. A reservoir plant for therapeutic applications: An overview. Pharmacogn Rev.

[11] Charles DJ, Simon JE (1992). A new geraniol chemotype of *Ocimum gratissimum* L. J Essential Oil Res.

[12] Obeng-Ofori D, Reichmuth C (1997). Bioactivity of eugenol, a major component of essential oil of *Ocimum suave* (Wild.) against four species of stored-product Coleoptera. Int J Pest Manag.

[13] Werker E, Putievsky E, Ravid U, Dudai N, Katzir I (1993). Glandular hairs and essential oil in developing leaves of *Ocimum basilicum* L. (Lamiaceae). Ann Bot.

[14] Siddiqui BS, Bhatti HA, Begum S, Perwaiz S (2012). Evaluation of the antimycobacterium activity of the constituents from *Ocimum basilicum* against *Mycobacterium tuberculosis*. J Ethnopharmacol.

[15] El-Beshbishy H, Bahashwan S (2012). Hypoglycemic effect of basil (*Ocimum basilicum*) aqueous extract is mediated through inhibition of α-gl*ucosidase and* α*-a*mylase activities: an in vitro study. Toxicol Ind Health.

[16] Parkinson J, Blaxter M (2009). Expressed sequence tags: An overview. Methods Mol Biol.

[17] Nagalakshmi U, Wang Z, Waern K, Shou C, Raha D, Gerstein M, Snyder M (2008). The transcriptional landscape of the yeast genome defined by RNA sequencing. Science.

[18] Collins LJ, Biggs PJ, Voelckel C, Joly S (2008). An approach to transcriptome analysis of non-model organisms using short-read sequences. Genome Inform.

[19] Lal RK, Khanuja SPS, Agnihotri AK, Shasany AK, Naqvi AA, Dwivedi S, Mishra HO, Dhawan OP, Kalra A, Singh A, Bahl JR, Singh S, Patra DD, Agarwal S, Darokar MP, Gupta AK, Gupta ML, Chandra R (2004). An early, short duration, high essential oil, methyl chavicol and linalool yielding variety of Indian basil (*Ocimum basilicum*) ‘CIM-Saumya’. J Med Arom Plant Sci.

[20] Wang Z, Fang B, Chen J, Zhang X, Luo Z, Huang L, Chen X, Li Y (2010). De novo assembly and characterization of root transcriptome using Illumina paired-end sequencing and development of cSSR markers in sweetpotato (*Ipomoea batatas*). BMC Genomics.

[21] Annadurai RS, Neethiraj R, Jayakumar V, Damodaran AC, Rao SN, Katta MAVSK, Gopinathan S, Sarma SP, Senthilkumar V, Niranjan V, Gopinath A, Mugasimangalam RC (2013). De Novo transcriptome assembly (NGS) of *Curcuma longa* L. rhizome reveals novel transcripts related to anticancer and antimalarial terpenoids. PLoS One.

[22] Kim HA, Lim CJ, Kim S, Choe JK, Jo S-H, Baek N, Kwon S-Y (2014). High-Throughput Sequencing and De Novo assembly of *Brassica oleracea* var. *Capitata* L. for transcriptome Analysis. PLoS One.

[23] An J, Shen X, Ma Q, Yang C, Liu S, Chen Y (2014). Transcriptome profiling to discover putative genes associated with paraquat resistance in goosegrass (*Eleusine indica* L.). PLoS One.

[24] Gahlan P, Singh HR, Shankar R, Sharma N, Kumari A, Chawla V, Ahuja PS, Kumar S (2012). De novo sequencing and characterization of *Picrorhiza kurrooa* transcriptome at two temperatures showed major transcriptome adjustments. BMC Genomics.

[25] Yang L, Ding G, Lin H, Cheng H, Kong Y, Wei Y, Fang X, Liu R, Wang L, Chen X, Yang C (2013). Transcriptome analysis of medicinal plant *Salvia miltiorrhiza* and identification of genes related to tanshinone biosynthesis. PLoS One.

[26] Lal RK, Khanuja SPS, Agnihotri AK, Misra HO, Shasany AK, Naqvi AA, Dhawan OP, Kalra A, Bahl JR, Darokar MP (2003). High yielding eugenol rich oil producing variety of *Ocimum sanctum* – CIM-Ayu. J Med Arom Plant Sci.

[27] Nagegowda DA (2010). Plant volatile terpenoid metabolism: Biosynthetic genes, transcriptional regulation and subcellular compartmentation. FEBS Lett.

[28] Jager S, Trojan H, Kopp T, Laszczyk MN, Scheffler A (2009). Pentacyclic triterpenoid distribution in various plants – rich sources for a new group of multi-potent plant extracts. Molecules.

[29] Razborsek MI, Voncina DB, Dolecek V, Voncina E (2008). Determination of oleanolic, betulinic and ursolic acid in lamiaceae and mass spectral fragmentation of their trimethylsilylated derivatives. Chromatographia.

[30] Huang L, Li J, Ye H, Li C, Wang H, Liu B, Zhang Y (2012). Molecular characterization of the pentacyclic triterpenoid biosynthetic pathway in *Catharanthus roseus*. Planta.

[31] Misra RC, Maiti P, Chanotiya CS, Shanker K, Ghosh S (2014). Methyl jasmonate-elicited transcriptional responses and pentacyclic triterpenoid biosynthesis in sweet basil. Plant Physiol.

[32] Kiferle C, Lucchesini M, Mensuali-Sodi A, Maggini R, Raffaelli A, Pardossi A (2011). Rosmarinic acid content in basil plants grown in vitro and in hydroponics. Cent Eur J Biol.

[33] Coon MJ (2005). Cytochrome P450: nature’s most versatile biological catalyst. Annu Rev Pharmacol Toxicol.

[34] Meijer AH, Souer E, Verpoorte R, Hoge JHC (1993). Isolation of cytochrome P450 cDNA clones from the higher plant *Catharanthus roseus* by a PCR strategy. Plant Mol Biol.

[35] Gupta P, Goel R, Pathak S, Srivastava A, Singh SP, Sangwan RS, Asif MH, Trivedi PK, Sangwan RS, Asif MH, Trivedi PK (2013). *De novo* assembly, functional annotation and comparative analysis of *Withania somnifera* leaf and root transcriptomes to identify putative genes involved in the withanolides biosynthesis. PLoS One.

[36] Berim A, Gang DR (2013). The roles of a flavone 6-hydroxylase and 7-O-demethylation in the flavone biosynthetic network of sweet basil. J Biol Chem.

[37] Fukushima EO, Seki H, Ohyama K, Ono E, Umemoto N, Mizutani M, Saito K, Muranaka T (2011). CYP716A Subfamily Members are Multifunctional Oxidases in Triterpenoid Biosynthesis. Plant Cell Physiol.

[38] Yuan L, Perry SE (2011). Plant Transcription Factors. Meth Protocol Mol Biol.

[39] Naika M, Shameer K, Mathew OK, Gowda R, Sowdhamini R (2013). STIFDB2- An updated version of plant stress-responsive transcription factor database with additional stress signals, stress-responsive transcription factor binding sites and stress-responsive genes in *Arabidopsis* and rice. Plant Cell Physiol.

[40] Colquhoun TA, Kim JY, Wedde AE, Levin LA, Schmitt KC, Schuurink RC, Clark DG (2011). *PhMYB4* fine-tunes the floral volatile signature of *Petunia*X*hybrida* through *PhC4H*. J Exp Bot.

[41] Spitzer-Rimon B, Farhi M, Albo B, Cna’ani A, Ben Zvi MM, Masci T, Edelbaum O, Yu Y, Shklarman E, Ovadis M, Vainstein A (2012). The R2R3-MYB-like regulatory factor EOBI, acting downstream of EOBII, regulates scent production by activating ODO1 and structural scent-related genes in petunia. Plant Cell.

[42] Suttipanta N, Pattanaik S, Kulshrestha M, Patra B, Singh SK, Yuan L (2011). The transcription factor CrWRKY1 positively regulates the terpenoid indole alkaloid biosynthesis in *Catharanthus roseus*. Plant Physiol.

[43] Li J, Blue R, Zeitler B, Strange TL, Pearl JR, Huizinga DH, Evans S, Gregory PD, Urnov FD, Petolino JF (2013). Activation domains for controlling plant gene expression using designed transcription factors. Pl Biot J.

[44] Mishra S, Triptahi V, Singh S, Phukan UJ, Gupta MM, Shanker K, Shukla RK (2013). Wound induced tanscriptional regulation of benzylisoquinoline pathway and characterization of wound inducible PsWRKY transcription factor from *Papaver somniferum*. PLoS One.

[45] Gong Z, Yamagishi E, Yamazaki M, Saito K (1999). A constitutively expressed Myc-like gene involved in anthocyanin biosynthesis from *Perilla frutescens*: molecular characterization, heterologous expression in transgenic plants and transactivation in yeast cells. Plant Mol Biol.

[46] Wang W, Jiang X, Zhang L, Chen P, Shen Y, Huang L (2011). Isolation and characteristic of SmbHLH1 gene in *Salvia miltiorrhiza*. Zhongguo Zhong Yao Za Zhi.

[47] Jin JP, Zhang H, Kong L, Gao G, Luo JC (2014). PlantTFDB 3.0- a portal for the functional and evolutionary study of plant transcription factors. Nucleic Acids Res.

[48] Darlington CD, Wylie AP (1995). Chromosome atlas of flowering plants.

[49] Mehra PN, Gill LS (1972). Cytology of west Himalayan Labiatae, tribe Ocimoideae. Cytologia.

[50] Carovic’-Stanko K, Liber Z, Besendorfer V, Javornik B, Bohanec B, Kolak I, Satovic Z (2010). Genetic relations among basil taxa (*Ocimum* L.) based on molecular markers, nuclear DNA content, and chromosome number. Plant Syst Evol.

[51] Lavania UC, Srivastava S, Lavania S, Basu S, Misra NK, Mukai Y (2012). Autopolyploidy differentially influences body size in plants, but facilitate enhanced accumulation of secondary metabolites, causing increased cytosine methylation. Plant J.

[52] Carels N, Hatey P, Jabbari K, Bernardi G (1998). Compositional properties of homologous coding sequences from plants. J Mol Evol.

[53] Vinogradov AE (2003). DNA helix: the importance of being GC-rich. Nucleic Acids Res.

[54] Wei W, Qi X, Wang L, Zhang Y, Hua W, Li D, Lv H, Zhang X (2011). Characterization of the sesame (*Sesamum indicum* L.) global transcriptome using Illumina paired-end sequencing and development of EST-SSR markers. BMC Genomics.

[55] Verma P, Shah N, Bhatia S (2013). Development of an expressed gene catalogue and molecular markers from the de novo assembly of short sequence reads of the lentil (*Lens culinaris* Medik.) transcriptome. Plant Biotechnol J.

[56] Le Dantec L, Chagné D, Pot D, Cantin O, Garnier-Géré P, Bedon F, Frigerio JM, Chaumeil P, Léger P, García V, Laigret F, De Daruvar A, Plomion C (2004). Automated SNP detection in expressed sequence tags: statistical considerations and application to maritime pine sequences. Plant Mol Biol.

[57] Zerbino DR, Birney E (2008). Velvet: Algorithms for de novo short read assembly using de Bruijn graphs. Genome Res.

[58] Schulz MH, Zerbino DR, Vingron M, Birney E (2012). Oases: Robust de novo RNA-seq assembly across the dynamic range of expression levels. Bioinformatics.

[59] Fu L, Niu B, Zhu Z, Wu S, Li W (2012). CD-HIT: accelerated for clustering the next generation sequencing data. Bioinformatics.

[60] Ashburner M, Ball CA, Blake JA, Botstein D, Butler H, Cherry JM, Davis AP, Dolinski K, Dwight SS, Eppig JT, Harris MA, Hill DP, Issel-Tarver L, Kasarskis A, Lewis S, Matese JC, Richardson JE, Ringwald M, Rubin GM, Sherlock G (2000). Gene ontology: tool for the unification of biology. Nat Genet.

[61] Moriya Y, Itoh M, Okuda S, Yoshizawa AC, Kanehisa M (2007). KAAS: an automatic genome annotation and pathway reconstruction server. Nucleic Acids Res.

[62] Langmead B, Trapnell C, Pop M, Salzberg SL (2009). Ultrafast and memory-efficient alignment of short DNA sequences to the human genome. Genome Biol.

[63] Mortazavi A, Williams BA, McCue K, Schaeffer L, Wold B (2008). Mapping and quantifying mammalian transcriptomes by RNA-Seq. Nat Methods.

[64] Anders S, Huber W (2010). Differential expression analysis for sequence count data. Genome Biol.

[65] Rastogi S, Kumar R, Chanotiya CS, Shanker K, Gupta MM, Nagegowda DA, Shasany AK (2013). 4-coumarate: CoA ligase partitions metabolites for eugenol biosynthesis. Plant Cell Physiol.

[66] Rada M, Ruiz-Gutiérrez V, Guinda Á (2011). Determination of Triterpenic Acids in Human Serum by High-Performance Liquid Chromatography- Triterpenoid Interaction with Serum Protein. J Agri Food Chem.

[67] Olofsson L, Engström A, Lundgren A, Brodelius PE (2011). Relative expression of genes of terpenoid metabolism in different tissues of *Artemisia annua* L. BMC Plant Biol.

