# Supplementary material for: De novo sequencing and comparative analysis of holy and sweet basil transcriptomes
Source: BMC Genomics. 2014 Jul 12;15(1):588. doi: 10.1186/1471-2164-15-588 (PMC4125705; doi:10.1186/1471-2164-15-588)

Additional file 1: Pathway assignment of *O. sanctum* and *O. basilicum*.unigenes based on Kyoto Encyclopedia of Genes and Genomes (KEGG).

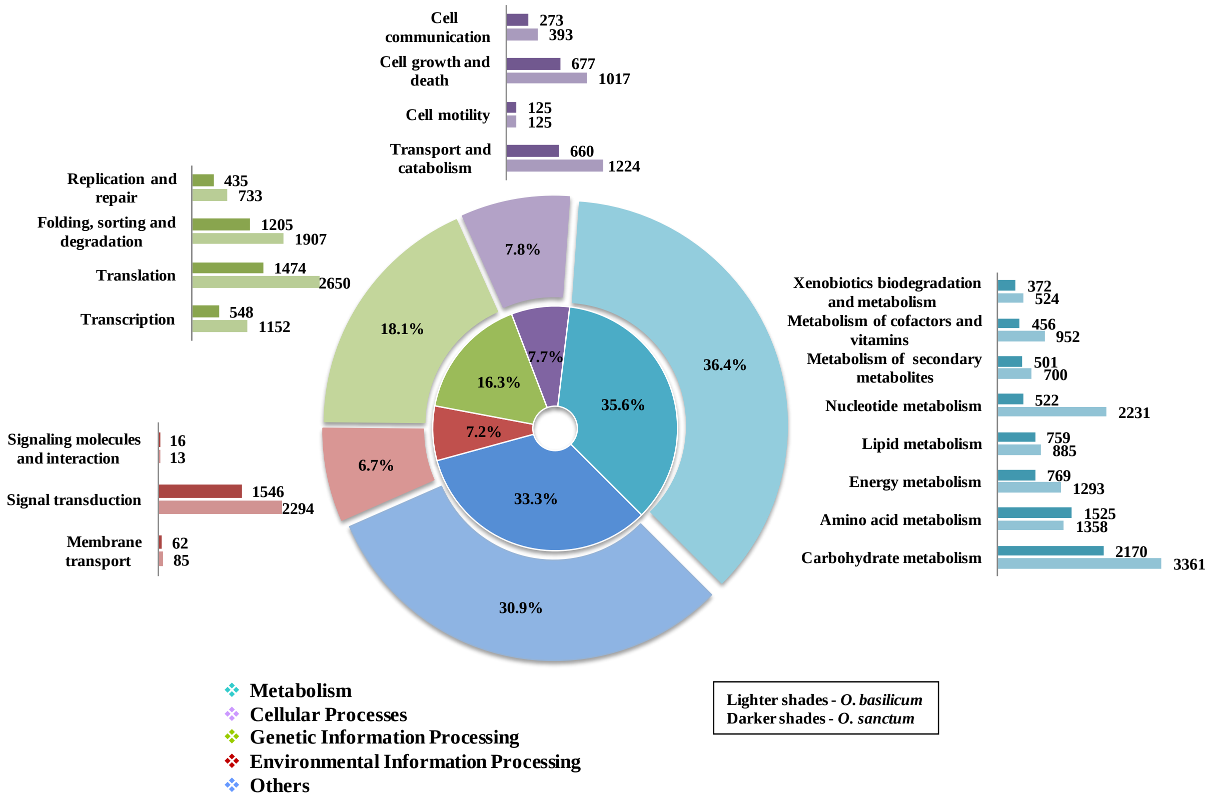

Supplement: Supplementary file 4 — Additional file 4: Pathway assignment of O. sanctum and O. basilicum. unigenes based on Kyoto Encyclopedia of Genes and Genomes (KEGG). (PDF 124 KB) [file 12864_2014_6319_MOESM4_ESM.pdf]
