# Supplementary material for: De novo sequencing and comparative analysis of holy and sweet basil transcriptomes
Source: BMC Genomics. 2014 Jul 12;15(1):588. doi: 10.1186/1471-2164-15-588 (PMC4125705; doi:10.1186/1471-2164-15-588)
Supplement: Supplementary file 6 — Additional file 6: Somatic chromosome preparations of (a) O. basilicum (2n = 48) and (b) O. sanctum (2n = 16) on a scale of 1 μm. (PDF 111 KB) [file 12864_2014_6319_MOESM6_ESM.pdf]

Additional file 2: Somatic chromosome preparations of (a) *O. basilicum* (2n=48) and (b) *O. sanctum* (2n= 16) on a scale of 1µm.

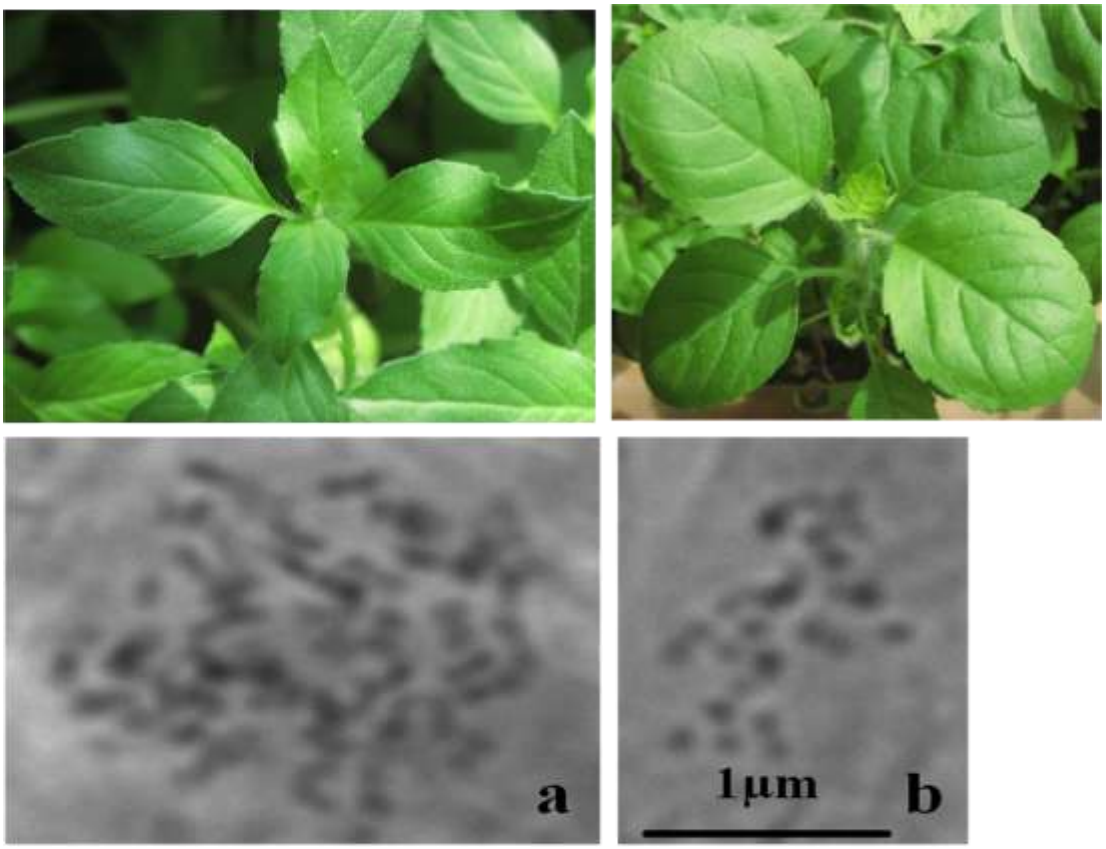

O. basilicum
(2n= 48)

O. sanctum
(2n= 16)
