# Supplementary figures and images for: De novo sequencing and comparative analysis of holy and sweet basil transcriptomes
Source: BMC Genomics. 2014 Jul 12;15(1):588. doi: 10.1186/1471-2164-15-588 (PMC4125705; doi:10.1186/1471-2164-15-588)

**Additional file 3:** Base composition report of *O. sanctum* and *O. basilicum*.

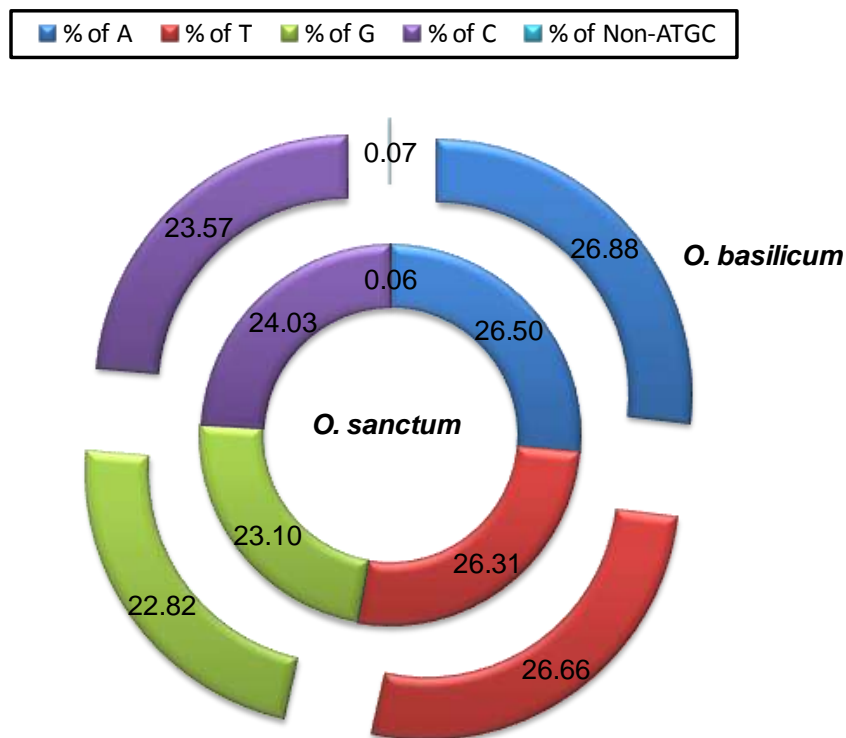

Supplement: Supplementary file 7 — Additional file 7: Base composition report of O. sanctum and O. basilicum. (PDF 138 KB) [file 12864_2014_6319_MOESM7_ESM.pdf]
